# Artificial intelligence in orthopaedics should be built with surgeons, not just for them!

**DOI:** 10.1002/jeo2.70920

**Published:** 2026-09-30

**Authors:** Felix C. Oettl, Balint Zsidai, Jacob F. Oeding, Ting Cong, Elmar Herbst, Gergely Pánics, Philipp W. Winkler, Volker Musahl, Eric Hamrin Senorski, Romain Seil, Christophe Ley, Yinan Yu, Thomas Tischer, Stefano Zaffagnini, Umile Giuseppe Longo, Michael T. Hirschmann, Michael Hantes, Kristian Samuelsson, ESSKA AI Committee

**Affiliations:** ^1^ Department of Orthopedic Surgery University Hospital Balgrist Zurich Switzerland; ^2^ Sahlgrenska Sports Medicine Center Gothenburg Sweden; ^3^ Department of Orthopaedics, Institute of Clinical Sciences, Sahlgrenska Academy University of Gothenburg Gothenburg Sweden; ^4^ Department of Orthopaedic Surgery University of Pittsburgh Pittsburgh Pennsylvania USA; ^5^ Gelenkpunkt‐Sports and Joint Surgery FIFA Medical Centre of Excellence‐Austria Innsbruck Austria; ^6^ Budapesti Uzsoki Street Hospital Budapest Hungary; ^7^ Department of Traumatology Semmelweis University Budapest Hungary; ^8^ FIFA Medical Centre of Excellence Budapest Hungary; ^9^ Department for Orthopaedics and Traumatology Kepler University Hospital GmbH, Johannes Kepler University Linz Linz Austria; ^10^ Department of Health and Rehabilitation, Institute of Neuroscience and Physiology Sahlgrenska Academy University of Gothenburg Gothenburg Sweden; ^11^ Department of Orthopaedic Surgery Centre Hospitalier de Luxembourg Luxembourg City Luxembourg; ^12^ Department of Mathematics University of Luxembourg Esch‐sur‐Alzette Luxembourg; ^13^ Department of Computer Science and Engineering Chalmers University of Technology Gothenburg Sweden; ^14^ Center for Digital Health Sahlgrenska University Hospital Gothenburg Sweden; ^15^ Department of Orthopaedic Surgery University Medicine Rostock Rostock Germany; ^16^ Department of Orthopaedic and Trauma Surgery Malteser Waldkrankenhaus Erlangen Erlangen Germany; ^17^ II Orthopaedic and Traumatologic Clinic IRCCS Istituto Ortopedico Rizzoli Bologna Italy; ^18^ Fondazione Policlinico Universitario Campus Bio‐Medico Roma Italy; ^19^ Research Unit of Orthopaedic and Trauma Surgery, Department of Medicine and Surgery Università Campus Bio‐Medico di Roma Roma Italy; ^20^ Department of Orthopaedic Surgery and Traumatology Kantonsspital Baselland Bruderholz Switzerland; ^21^ University of Basel Basel Switzerland; ^22^ Department of Orthopaedic Surgery and Musculoskeletal Trauma, Faculty of Medicine, School of Health Sciences University of Thessalia Larissa Greece; ^23^ Department of Orthopaedics Sahlgrenska University Hospital Mölndal Sweden

## Abstract

Surgical artificial intelligence (AI) is typically validated on one patient population and then deployed unchanged into another, without surgeon input into its design. AI in orthopaedics should be built with surgeons, not simply for them: Surgeons should hold shared authority over the clinical question, data curation, validation, implementation, monitoring and retirement of any tool they use. Specialty‐tuned models outperform general‐purpose systems on some tasks, but the margin is modest and likely to narrow as generalist models improve. The more durable distinction is who controls the update loop—who can retrain a model on local outcomes, audit its failures and withdraw it when it drifts. Trial evidence shows that a clinician kept in that loop by design, not as an afterthought, outperforms working alone. What determines whether orthopaedic AI fulfils its promise is not model architecture, but who stays accountable to the surgeon at the bedside when the model needs to change.

**Abbreviations**
AI: artificial intelligence
AIME: Articulate Medical Intelligence Explorer
AUC‐ROC: area under the curve—receiver operating characteristic
ESSKA: European Society of Sports Traumatology, Knee Surgery and Arthroscopy

Consider a diagnostic model validated on a cohort in Boston and deployed unchanged into a trauma unit in Zurich, treating a patient population and case mix based on an imaging protocol it has never seen in training. This is not a hypothetical; it is the default path most surgical artificial intelligence (AI) takes from validation study to clinical use.

Surgeons are rarely consulted before a tool reaches them, and patients notice the gap even when surgeons do not name it directly: Trust within the clinical relationship can erode when a third party—one that neither clinician nor patient helped shape—begins making recommendations [11]. Put simply, clinical trust is established between patients and clinicians—not with an external, third‐party system over whose development neither has had influence.

A systematic review of deep learning studies claiming clinician‐level performance found that most were designed and reported too poorly to support the comparison being made [10]. The problem is not that these models are unsophisticated. It is that they were built for surgeons rather than with them, and the difference primarily becomes evident in trust, then in adoption and finally in outcomes.

The European Society of Sports Traumatology, Knee Surgery and Arthroscopy (ESSKA) AI Committee has been making a version of this argument for a year without naming it as a single thesis. We have written about the difference between de‐skilling and up‐skilling, arguing that AI should be positioned to liberate clinicians from repetitive tasks rather than to erode the judgement underneath them [13].

It is the purpose of this editorial to state the underlying claim directly: AI in orthopaedics should be built with surgeons, not just for them. In practice, this means *with* surgeons rather than *for* them, including shared design authority across the lifecycle—from defining the clinical question and curating data to validation, implementation, monitoring and retirement.

Surgical judgement resists full codification. It is built from repeated exposure to cases that do not fit the textbook, and it updates continuously as a surgeon's caseload changes. A model trained once on a static dataset cannot absorb this kind of learning after deployment, which is one reason a *JAMA Surgery* review called for AI to augment surgical decision‐making rather than to formalize it into a fixed protocol and warned explicitly against losing bedside assessment and human intuition in the process [8]. A more recent state‐of‐the‐art review of AI‐enabled surgical decision support reaches a similar conclusion from the engineering side: the systems that perform best in practice are the ones designed around continuous surgeon interaction, not the ones designed to replace a step in the workflow outright [7].

Specialty‐specific, fine‐tuned models may outperform general‐purpose systems on clinically relevant tasks within a given department, particularly with respect to efficiency. The ESSKA AI Committee advanced a similar argument last year regarding small language models tailored to orthopaedic practice [14]. A 2025 study fine‐tuned a pathology foundation model for lung cancer biomarker detection using real deployment data and demonstrated performance gains that a frozen, off‐the‐shelf version of the same model could not match [2]. A large language model built specifically for cardiology care outperformed generalist alternatives on the reasoning tasks cardiologists actually face [12]. But we should not overstate how durable this advantage is architecturally.

However, the durability of this advantage should not be overstated. A 2026 comparative evaluation of disease‐risk prediction using structured electronic health records found that fine‐tuned specialist clinical foundation models exceeded fine‐tuned generalist language models by less than one percentage point in area under the curve—receiver operating characteristic (AUC‐ROC) curve. Moreover, embeddings generated by general‐purpose models compared with a simple classifier matched or exceeded the performance of both approaches, while requiring substantially lower engineering effort [9]. Although these findings remain preprint evidence and are inherently task‐dependent, they provide an important corrective: Specialization is not an immutable principle in medical AI. Rather, it currently represents a modest advantage that is likely to diminish as increasingly capable generalist models continue to evolve.

This reframes the argument. Google's Med‐PaLM demonstrated that large language models can encode a striking amount of clinical knowledge [16], and its successor, Articulate Medical Intelligence Explorer (AMIE), has shown genuine promise as a conversational diagnostic system [18]. These are real achievements, not rivals to dismiss. The distinction that matters is not whether a model is large and general or small and specialized. It is who holds the update loop: who can retrain it on local outcomes, audit its failures and revoke its use when it drifts.

Med‐PaLM and AMIE were trained centrally and remain under their maker's control; a department that adopts them adopts someone else's judgement about what the model should know next. Open‐weight alternatives such as DeepSeek‐R1 show that frontier‐level reasoning does not require surrendering that control [3], and the goal for orthopaedic AI is to demand the same adaptation rights from any vendor, not to reject powerful models on principle. In practice, procurement should define access to local performance and subgroup metrics, version and data‐provenance documentation, responsibilities for recalibration or retraining, human‐factors testing, incident reporting and pre‐agreed criteria for suspension or rollback. Any material adaptation must remain within an approved change‐control, quality‐management, ethical and regulatory pathway. None of this requires rejecting a capable vendor. It requires refusing opacity about performance, change and accountability as the price of capability.

A recent in silico evaluation of locally deployed language models answering postoperative patient questions found that open, locally hosted models still lag commercial ones on safety‐relevant outcomes, including fabrication and harmful content [5]. Local is not a synonym for safe; it is a precondition for accountability, contingent on investment in fine‐tuning, validation and guardrails that most departments have not yet made. A recent analysis frames deployment as a moving‐target problem: Both the model and the clinical environment it serves continue to change after go‐live, so validation cannot be a single gate passed once [6]. The ask is not that every department trains its own model from scratch. It is that whoever controls the update loop, department or vendor, is answerable to the surgeons using the output.

Most orthopaedic departments do not have the engineering capacity to hold that loop alone and pretending otherwise would repeat the same overreach this editorial argues against. That is precisely the gap a professional society exists to fill: Pooling validation data across member institutions, negotiating audit rights collectively that no single department could win alone and maintaining shared infrastructure over years rather than a single grant cycle.

Prospective trial data show that keeping a human in that loop, built in by design rather than added as a hedge, produces measurable benefits. A recent trial found that outpatient reception handled jointly by nurses and a large language model outperformed them working independently [19] and a proof‐of‐concept trial of reinforcement learning for glycaemic control in Type 2 diabetes reached a comparable conclusion [20]. None of these systems succeeded in minimizing the clinician's role. They succeeded by giving the clinician a durable, monitored role to play.

Such partnerships cannot rely on goodwill alone, nor can they be established through a single implementation effort. Participatory design and lifecycle accountability require governance infrastructure: reporting standards, staged clinical evaluation, external and subgroup validation, surveillance for calibration and fairness drift and explicit routes for rollback or withdrawal. A 2024 framework highlighted the risk of AI misuse in biomedical research [17] and MedHELM, published this year, offers a holistic evaluation standard for medical language models that goes beyond accuracy on isolated benchmarks [1]. Orthopaedic AI needs its own version of this infrastructure and needs to plan for maintaining it, not just publishing it once.

The barrier to prototyping such tools has fallen sharply: what once required a team of engineers working for months can now be sketched by a single clinician in hours, a shift this Committee has argued should be met with disciplined validation rather than reflexive resistance [15]. That is a claim about prototyping speed, not about deployment. The barriers that matter most, validation, safety and who is accountable when a model is wrong, have not fallen at all, and no one should pretend otherwise.

Seven years ago, a review called AI in surgery a matter of promises and perils in roughly equal measure [4]. That balance was never going to be determined solely by technological progress or by the location in which a model happened to be trained. Ultimately, it will be determined by who controls the update cycle and who remains accountable to the surgeon at the bedside when the model inevitably needs to change.

## FUNDING INFORMATION

The authors have no funding to report.

## CONFLICT OF INTEREST STATEMENT

Kristian Samuelsson is a member of the Board of Directors of Getinge AB (publ) and a medtech advisor to Carl Bennet AB. The remaining authors declare no conflicts of interest.

## ETHICS STATEMENT

The authors have nothing to report.
